# Challenges of web-based personal genomic data sharing

**DOI:** 10.1186/s40504-014-0022-7

**Published:** 2015-03-27

**Authors:** Mahsa Shabani, Pascal Borry

**Affiliations:** Centre for Biomedical Ethics and Law, Department of Public Health and Primary Care, University of Leuven, Kapucijnenvoer 35, Box 7001, 3000 Leuven, Belgium

## Abstract

In order to study the relationship between genes and diseases, the increasing availability and sharing of phenotypic and genotypic data have been promoted as an imperative within the scientific community. In parallel with data sharing practices by clinicians and researchers, recent initiatives have been observed in which individuals are sharing personal genomic data. The involvement of individuals in such initiatives is facilitated by the increased accessibility of personal genomic data, offered by private test providers along with availability of online networks. Personal webpages and on-line data sharing platforms such as *Consent to Research (Portable Legal Consent)*, *Free the Data*, and *Genomes Unzipped* are being utilized to host and share genotypes, electronic health records and family history uploaded by individuals. Although personal genomic data sharing initiatives vary in nature, the emphasis on the individuals’ control on their data in order to benefit research and ultimately health care has seen as a key theme across these initiatives. In line with the growing practice of personal genomic data sharing, this paper aims to shed light on the potential challenges surrounding these initiatives. As in the course of these initiatives individuals are solicited to individually balance the risks and benefits of sharing their genomic data, their awareness of the implications of personal genomic data sharing for themselves and their family members is a necessity. Furthermore, given the sensitivity of genomic data and the controversies around their complete de-identifiability, potential privacy risks and harms originating from unintended uses of data have to be taken into consideration.

**Electronic supplementary material:**

The online version of this article (doi:10.1186/s40504-014-0022-7) contains supplementary material, which is available to authorized users.

## Introduction

The decreasing costs of sequencing technologies (Hayden 2014) supported by recent advancements in bioinformatics amounted to an improvement in genomic data production. In light of the increasing availability of genomic data, data sharing and open access policies were promoted by large international consortia in order to maximize the use of generated datasets and enhance the statistical power of studies. Funding organizations also drafted policies to expand accessibility and use of datasets by requiring researchers to incorporate data sharing plans in their fund-seeking proposals (Foster and Sharp 2007; NIH 2014). As a consequence, central on-line databases such as the database of Genotypes and Phenotypes (dbGaP)^a^ and the European Genome-phenome Archive^b^ have been designated to host vast volumes of data either in a publicly accessible or controlled fashion.

Not surprisingly, the unique features of data sharing challenged the governance of genomic databases (Knoppers et al. 2007; Peppercorn et al. 2012). The subsequent use of genomic data originally accumulated for a single project gives a new twist to the discussion on the adequacy of informed consent, and the need to inform research participants about potential risks, benefits, research goals and withdrawal options. This developments made some to conclude that in the age of data sharing, traditional paper-based one-off consent is stretched to its limits (Hayden 2012). Additionally, ensuring privacy of participants appears as an emerging challenge. Although de-identification of data has been traditionally seen as a response to privacy concerns, it might fall short in the context of genomic data (Kaye 2012), given that DNA is a unique identifier.

Although responsibilities of researchers and biobanks in fostering sharing practices are extensively pronounced, also individuals are appealed to contribute to such initiatives (Dolgin 2010; Hand 2010; Kaye 2012). Web-based personal genomic data sharing or “crowdsourcing” research (Barry 2009) initiatives encourage the provision of personal genomic data and health-related information by individuals for research purposes. The involvement of individuals in such personal data sharing initiatives is facilitated by the increased accessibility of personal genomic data, offered by commercial test providers along with the availability of interactive information technology. Promises to utilize potentials of personal genomic data motivate individuals to take part in sharing activities.

Various personal genomic data sharing initiatives are being organized differently, according to their missions and goals. In some occasions, personal genomic data sharing is epitomized in the form of posting personal genomic data or those of family members at publicly accessible personal webpages. For example, the idea of Corpasome refers to an effort by Corpas family to publicly share the personal genomic data belonging to five members of the family (Corpas 2013). In these situations personal genomic data is usually posted in order to broaden access and enable subsequent analysis and interpretation by various interested researchers.

In other occasions, intermediary data sharing platforms may facilitate the personal genomic data sharing by initiating projects to host and share genotype data and medical information. Thereby, individuals who have access to their genomic data and medical records can upload their data into the designated webpages. *Free the data*^c^, *Genomera*^d^ and *PatientsLikeMe*^e^ are examples of these projects. In the framework of these platforms, data collection and sharing policies have often been delineated within the privacy policy statements or consent forms. In addition, in some projects such as *Free the Data*, participants are asked to take part in the surveys and to answer various questions regarding their health status or any existing health conditions in the family history.

Despite the observed differences, the emphasis on the individuals’ control on their data and the possibility of sharing data have been seen as a key theme across these initiatives. To enable individuals to control their personal data, customized sharing options have been provided to participants. Such strategy was designed with the aim to respond to the inadequacy of employing one-size-fits-all approach towards consent and privacy preferences (Nuffield Council on Bioethics 2013). Thereby, individuals are asked to determine who should have access to their data and for which purposes (Kaye et al. 2013).

Additionally, it has been argued that an ongoing engagement of individuals enabled by web-based tools may address a challenge of maintaining contact with participants (Wee 2013). As individuals could opt to be re-contacted by third party researchers in the course of personal data sharing, collection of more detailed real-time information upon the request of researchers has been advanced as a plausible practice (McEwen et al. 2013). Such “dynamic phenotyping” (Lunshof et al. 2010) supported by web-based personal genetic data sharing has been presented as a potential response to the need for linking and collecting phenotypic and other health related information in longitudinal prospective studies in which genomic data are generated.

Ethical and legal dilemmas faced by researcher-driven or biobank-driven genomic data sharing have been extensively discussed in the literature (Fullerton et al. 2010; Harris et al. 2012; Kaye et al. 2009; Knoppers et al. 2011). A few studies also have reflected on attitudes of consumers concerning sharing personal genomic data (Lee et al. 2013) and policies employed by direct-to-consumer genetic testing companies regarding their research activities fueled by consumer data (Esposito and Goodman 2009; Harris et al. 2013; Howard et al. 2010). However, little has been published about the ethical and legal challenges surrounding growing web-based personal genomic data sharing (Bloss 2013; Angrist 2014; Mathews and Jamal 2014). To bridge this gap, this paper investigates the challenges surrounding such initiatives in view of three examples of current web-based personal genomic data sharing projects, namely *Consent to research (Portable Legal Consent)*^f^, *Free the Data*^c^*and Genomes Unzipped (GNZ)*^g^ (Table 1). Two ultimate goals will guide our study. Firstly, we aim to analyze the legal and ethical dilemmas linked with personal genomic data sharing and assess existing safeguards. Secondly, we aim to provide insight into how these challenges have been tackled in the framework of these three projects. To this end, the first part of this article highlights the main features of the three projects on the basis of consent forms, privacy policy statements, and relevant information retrieved between October 2013 and February 2014, from the website of these projects. In the second part, some concerns arising from personal genomic data sharing, in particular re-identification risks, legal safeguards and governance mechanisms, implications for family members, limitations of individuals’ control on their genomic data and potential responses of the three projects to these concerns, are discussed.

**Table 1:** **Features of three web-based personal genomic data sharing projects**

**Name of the project**	**Type of data they collect**	**Goals**
Consent to research (Portable Legal Consent)	exome, whole-genome sequence, electronic health records or family history	To create large datasets of user-contributed health and genomic data that are easier to re-use, to promote collaborations and innovation around health and genomic research
Free the data	Mutation reports for *BRCA1/2*, a variety of health information	To invite clinicians and individuals to donate their mutations and associated clinical data in order to be deposited in *ClinVar* and other open-access databases
Genomes unzipped (*GNZ)*	Genotypes obtained from various genetic test providers, release of more data up to and including whole-genome sequencing is envisioned	To use the data provided by participants and other *GNZ* community members both to perform scientific analyses and as a basis for communicating about genetics to a broader audience

### Web-based personal genomic data sharing projects: three examples

In order to shed light on the main features of the three web-based personal genomic data sharing projects under study, the consent form, privacy policy statements, and other relevant information extracted from the websites of these projects have been consulted. As indicated in the following, personal genomic data sharing practices share some similarities such as placing individuals in the control of their data and utilizing the web-based vehicles to facilitate personal data sharing, along with stressing the significance of such contribution on expediting research. But they also differ in various aspects including missions, scale, and privacy models.

### Consent to research

*Consent to Research* is a project initiated by *Sage Bionetworks*^h^, a non-profit research organization based in Seattle, U.S. which aims to redefine “biomedical research through open systems, incentives, and norms.” To accomplish these goals, *Sage Bionetworks* adopted the *Portable Legal Consent (PLC)*^i^ mechanism as a part of a governance project that includes terms of use, privacy policies, and data use agreements as well as informed consent and legal terms. As elaborated within the *PLC*, the project endeavors “to create large datasets of user-contributed health and genomic data that are easier to re-use, to promote collaborations and innovation around health and genomic research and to allow any volunteer to play an active role in the research process”. In pursuit of this mission, *Sage Bionetworks* collects genomic, phenotypic, observational, health-related and other forms of information from volunteer individuals, to feed multiple research studies. The first study of this project namely *Self-Contributed Cohort for Common Genetic Research (SCC-CGR)* is currently closed for enrollment and the publication of results and data (aggregate level) is expected in the near future^f^.

Upon participation in this project, individuals may agree to grant rights to research, redistribute, publish and commercialize their data to “Qualified Researchers”. According to the *PLC*: “Data collected will be made available, for research purposes, to a broad variety of users who have agreed to be bound by a contract and to specific terms and conditions that include use of the data in an ethical manner, to do no harm, not re-identify or re-contact individuals, and to make their research results openly available to the general public”^i^.

In addition, in an on-line educational video that cannot be bypassed, potential participants learn about the importance of wide data sharing to be materialized in an ethically sound fashion^j^. In terms of disclosure, *PLC* provides participants with an option to share their data in a de-identified fashion, unless otherwise desired. Additionally, a non-comprehensive list of potential risks and discomforts is incorporated within *PLC* about unintended uses of shared data by third parties that could consequently harm participants or their family members. The unintended uses of data have been further illustrated by a few examples such as using data in a discriminatory fashion against data subjects or their family members in the employment, insurance or financial services setting. In terms of benefits, it has been articulated that the contribution of personal data would not yield any direct benefit for the participants, given the fact that society and science at large are the conceived beneficiaries of such initiatives.

### Free the data

*Free the data*^c^ is a project initiated by a consortium of organizations, managed by *Genetic Alliance* which is a non-profit health advocacy organization. *Free the Data* aims to facilitate discovery of “association between mutations and health outcomes” via entering *BRCA1/2* reports into the public database of *ClinVar* which is a free, publicly accessible archive of reports and information on gene variants. Individuals who receive a report entailing a mutation in *BRCA1* or *BRCA2* gene are invited by *Free the Data* to provide a variety of personal health information and share their medical reports in a public database, in order to realize “a better understanding of disease, higher quality patient care, and improved human health”^k^. Besides personal medical reports, patients are also offered the options to upload reports from their legal minors or other family members to the website^l^ .For the latter, however, it is required to observe the wishes of family members over submission of their data in the website. In addition, in the framework of the *Sharing Clinical Reports Project* (SCRP) which is the clinician arm of the *Free the Data* project, clinicians could submit their patients’ *BRCA1/2* reports in a de-identified fashion^m^.

Individuals are enabled to indicate their sharing preferences towards a variety of potential data users grouped as “Advocacy and Support Groups” and “Medical Researchers”, and are able to modify their choices at any time (Nguyen and Terry 2013). According to the “About Us” webpage of the project, “Free the Data uses a participant-centric platform that allows you to decide how much you share, and with whom. Using privacy and security technology similar to banking, you have control over your health information and can set your own sharing, privacy, and data access preferences”^n^.

### Genomes unzipped

*Genomes Unzipped (GNZ)* is a project initiated by a group of active researchers in various fields of genetics, as well as specialists in the legal and public health issues surrounding new genomic technologies. In this project, members of *GNZ* publicly publish their own genomic data believing that “doing good science means releasing complete data for others to investigate”^g^. Generating “tools for analysis of raw genetic data” along with “providing independent, unbiased assessments of the technical validity and clinical utility of a variety of genetic testing products” are included in the perceived outcomes of the project within the *Participant Information Form*.

Additionally, by endorsing large open-access non-anonymous research databases such as the *Personal Genome Project*^o^, *GNZ* encourages individuals to participate in an open research project in the benefit of the public good. Although, at this moment participation of *GNZ* is limited to 15–20 core members who have expertise in an area related to the analysis of genetic data and their family members, it has been envisioned that individuals from the broader community who have access to their genetic data will be invited to share their data in the future. According to the webpage of *GNZ* project, the members of the project hope that releasing their data publicly “will help to guide useful discussions about genetic privacy and the benefits, risks, and limitations of genetic information in general”, as they “believe that many of the fears expressed about the dangers of genetic information are exaggerated, and see this project as an opportunity to have a constructive public discussion about the truth behind these fears”^g^. *GNZ*, in tune with the ultimate goal of the project to reform the perception of genetic privacy, takes a rather different approach compared to the two other projects by informing participants that no level of privacy, anonymity or confidentiality is guaranteed. Similar to *PLC*, a list of potential risks and discomforts associated with personal genomic data sharing has been laid out in the *Participant Information Form* of *GNZ* project to be factored in the process of decision making by participants.

### Concerns related to web-based personal genomic data sharing projects

Activities of web-based personal genomic data sharing initiatives may raise a number of ethical and legal challenges. The following part addresses the paramount concerns associated with such initiatives and assesses the current responses of the studied projects to those concerns.

### Identifiability and Re-identification

The potential accessibility of linkable data augments the concerns of re-identification of shared personal genomic data. This was recently shown in a study (Gymrek et al. 2013) in which researchers could successfully recover surnames of five de-identified participants of the *1000 Genomes project*^p^ by profiling short tandem repeats on the Y chromosome and by querying recreational genetic genealogy databases. The integration of genomic data with health information and electronic medical records is another element that increases the possibility of re-identification of data subjects (Esposito and Goodman 2009).

In line with the privacy concerns and demonstrated risks of re-identification, the studied projects attempt to inform participants regarding privacy risks associated with broad personal genomic data sharing. According to *PLC*, although participants could opt-in for a de-identified data sharing, the risk of re-identification and loss of privacy due to erroneous or malicious identity disclosure should be considered. *Free the data* project also warns participants within the privacy policy statement about the impossibility of guaranteeing absolute privacy due to the fact that “new vulnerabilities and threats appear every day”. The project promises participants to “minimize risks to a tolerable level”, as it is not possible to eliminate data protection risks thoroughly. On the other hand, the *GNZ* project favors the non-anonymous personal data sharing approach, stating that “given the ease with which a dedicated snoop could obtain genetic information surreptitiously (via shed skin, hair or saliva, for instance), some of us argue that the whole notion of genetic privacy is illusory anyway – while releasing our data online makes it easier for people to get hold of it, this is a difference of degree rather than kind”^g^.

However, the implications of disclosure of re-identification risks by personal data sharing projects are matter of discussion. One can argue that by signing an open consent (Lunshof et al. 2008), encompassing, among others, potential risks of re-identification, participants implicitly relinquish their privacy and confidentiality rights over the shared data. According to the open consent model, de-identifiability of genomic data is a far-fetched promise, therefore participants should be prepared to embrace the risks concomitant with genomic data sharing (Lunshof et al. 2008).

Even if signing such a consent form removes potential legal responsibilities from the shoulder of data sharing platforms towards participants, this should not be seen as an ethical approval for re-identification attempts. In the aftermath of the recent re-identification demonstrations (Gymrek et al. 2013; Homer et al. 2008), concerns have been raised by, inter alia, the actual participants of personal genomic data sharing projects pointing out that the non-consensual re-identification attempts are ethically reprehensible (Meyer 2013). It has been argued that the consent of individuals to participate despite the potential risks of re-identification should not be construed as consenting to re-identification per se. “Even if the consent form signed at the time of the original collection includes a disclaimer that absolute anonymity cannot be guaranteed, re-identifying the DNA sample later represents a new collection, one that has been undertaken without any consent” (Wilson 2013). In essence, to re-identify individuals, shared genotypes (and associated data such as gender, age or disease being studied) should match against available reference genotypes which contain personal datasets with identifiers. Alternatively they should link to non-genetic databases such as data in health-care, administrative, criminal or disaster response databases (Lowrance and Collins 2007). Following either of these routes would result in associating de-identified personal data with variety of identifiable information, while the consent of data subjects is lacking.

Therefore, in order to respect rights of research participants, data sharing projects that engage in de-identified personal data sharing activities, should craft sufficient mechanisms to reduce the risks of re-identification in accordance with their data stewardship responsibilities. In addition, third party users should be warned against the ethical and potential legal repercussions of re-identification attempts due to the violation of participants’ rights.

### Legal safeguards and Governance mechanism

Besides the re-identification risks, sharing personal genomic data may incur risks for harms on participants. In this regard, *PLC* and *GNZ* canvas a list of potential harms stemming from publicly sharing personal genetic data, mentioning that the evolving nature of genomics research makes it impossible to predict the breadth of the risks or their magnitude. Some of the included risks for harms appear rather remote such as “synthesizing DNA strings and placing them at a crime scene” by malicious users. However, other examples such as discriminatory use of data against individuals or their family members by employers or insurance companies seem within the reach. Although the effort of data sharing projects in candidly informing participants about potential risks and discomforts is necessary, it still needs to be coupled with legal and ethical safeguards if harms occur (Prainsack and Buyx 2013).

The potential legal responses to the harms associated with personal genomic data sharing vary across jurisdictions. In terms of discriminatory uses of data, for instance, *PLC* and *GNZ* inform participants that existing legal protections may not be comprehensive. As it is stated within the *Participant Information Form (GNZ)*: “Although some countries have laws that prohibit certain forms of genetic discrimination, these laws may not apply to you, may not protect against all forms of discrimination or may not stop a third party from discrimination against you even where it is prohibited by law”. This may beg the question concerning the sufficiency and effectiveness of current legal and ethical safeguards in shielding participants against potential harms (Weil et al. 2013). This is crucial mainly because the existing legal and ethical privacy safeguards might fall short in the view of voluntarily sharing personal genomic data and medical records outside the confidential relationship of researchers-individuals. “[...] as patients become more empowered to share their data to achieve greater medical benefit from it, and as we move to more seamlessly map between DNA and more easily acquired high-dimensional phenotypic data to predict with greater ease a greater diversity of human behaviors and disease risks, laws must also evolve to ensure that the rights of patients are protected. The shift to a more open personal data environment and a greater participation of informed patients will thus need to be accompanied by stricter and broader anti-discrimination regulations” (Schadt 2012).

That said, initiating personal genomic data sharing projects despite the acknowledged shortcomings in current legal safeguards and governance and oversight mechanisms may raise concerns regarding protection of research participants.

### Personal genomic data sharing: Not an entirely personal decision

The hereditary nature of genomic data may transform the personal decision of sharing genetic data into one with familial ramifications. As in the *Participant Information Form, GNZ* puts it: “Although in many instances any conclusions that may be inferred from your publicly available information may be speculative with respect to you, and even less predictive with respect to your family members, the complete set and magnitude of the risks that the public availability of this information poses to you and your relatives are not known at this time”^q^. In particular, genomic data sharing by individuals may reveal a spectrum of unexpected health and non-health-related information with significance for immediate family members. For instance, by analyzing publicly available personal genetic data, third parties could unveil the elevated or diminished risks for a disease or other trait or shed light on the family lineage and ethnicity. As a result, this information “may alter how individuals view themselves and their family of origin” (Doukas and Berg 2001).

Also, in the framework of *PLC*, individuals are notified that the provided list of risks and discomforts could address both participants and their family members. The potential risks for relatives are further elaborated: “If a privacy breach results in the disclosure of information pertaining to an inherited mutation, for example, that information may harm not only the individual research participant but also other genetically related people. Under such circumstances, an individual’s choice to participate in research may result in group discrimination or stigmatization” (Weil et al. 2013).

In response to this concern, both *GNZ* and *PLC* strongly encourage participants to discuss the project and pertinent risks with their immediate family members. Notwithstanding, at this moment no mechanism exists to ensure that such consultation is conducted. Moreover, it is expected that the likely conflict of interests between family members concerning sharing genetic data would bring vexing issues of data ownership and scope of privacy rights to the fore and require further investigations.

### Limitations of individuals’ control on their genomic data

Placing individuals in control of their data is seen as a recurring theme across the studied projects. In line with this goal, participants are solicited to individually balance risks and benefits of personal genomic data sharing in congruence with their heterogeneous risk-benefit preferences.

To make an informed choice, individuals should be able to grasp the breadth and magnitude of potential risks such as privacy breaches. However, one may legitimately argue conducting such risk and benefit analysis requires holding sufficient knowledge concerning complex and yet unknown potentials of personal genomic data sharing, which general public may lack (Steinsbekk et al. 2013). “by building increasingly large databases and collecting both genetic and extensive phenotypic information, however, associations may later be made that were not clear or possible to predict, and these might constitute findings (about personality or behavior, for instance) that an individual might have wanted to protect or conceal” (Esposito and Goodman 2009).

Complexities of the information extracted from genomic data have been illustrated in the case of the public release of Dr. James Watson’s sequenced genome in 2008. In course of this public release, on the basis of Dr. Watson’s request, all the gene information about apolipoprotein E (ApoE) had been removed due to concerns regarding a shown association between this gene and late-onset Alzheimer’s disease (LOAD). Despite undertaking the precautionary measures, a subsequent study suggested the insufficiency of the deletion of the APOE gene information to prevent a risk prediction for LOAD: “the deletion of the APOE gene information only may not prevent accurate prediction of Dr. Watson’s risk for LOAD conveyed by APOE risk alleles. Specifically, linkage disequilibrium (LD) between one or multiple polymorphisms and APOE can be used to predict APOE status using advanced computational tools. Therefore, simply blanking out genotypes at known risk factors is generally not sufficient if the aim is to hide genetic information at these loci” (Nyholt et al. 2009).

The investigated personal genomic data sharing projects attempt to familiarize participants with the implications of their contribution through consent and the privacy policy statements. It is however unclear whether such documents are sufficient in informing individuals about potential risks of data sharing. Results of studies indicate that participants often have difficulty in comprehending lengthy consent forms, understanding confidentiality issues and recalling what they did consent for (Ormond et al. 2009; Robinson et al. 2013). Likewise, designing privacy policy statements to fulfill informational needs of participants and specify their privacy preferences has been seen to be a challenging effort. As Cranor et al. note, the underlying reasons for such challenges may include that “privacy policies are complex, user privacy preferences are often complex and nuanced, users tend to have little experience articulating their privacy preferences, users are generally unfamiliar with much of the terminology used by privacy experts, and users often do not understand the privacy-related consequences of their behavior” (Cranor et al. 2006).

Presumably, barriers in adequately informing individuals regarding personal data sharing led some projects to target highly educated or more motivated and empowered individuals. Alternatively, participants may be required to pass an “entrance test” before widely sharing their data, a model that employed by *Personal Genome Project* (Lunshof et al. 2008). However, this approach has been criticized on the grounds that the sample may not be representative of whole society as such, imposing a selection bias (Janssens and Kraft 2012).

To address the need for adequately informing participants, some personal genomic data sharing projects have referred to supplementary mechanisms. For instance, *GNZ* encourages participants to seek advice from their physician or other qualified health care providers concerning implications of the participation. However, a likely shortage of knowledge and experience of general health care professionals in genetics may hinder such consultation. In this regard, a survey of consumers of Direct-to-Consumer personal genomics indicated “the majority of participants reported that their healthcare provider had limited ability to understand and insufficient expertise to interpret their results” (Lee et al. 2013).

Furthermore, the option to withdraw data from the research is central to being in control of personal genomic data. However, sharing data through the web imposes significant barriers on a complete withdrawal of shared data and could make personal data sharing an irrevocable decision.

### Concluding remarks

The imperative to broad genetic data sharing grows stronger with time. Along with data sharing by researchers, various initiatives invite individuals to participate in personal genomic data sharing. In those initiatives, individuals are encouraged to take control of their genetic data and utilize web-based tools to share their data according to their privacy comfort. It has been argued that by enabling such an informed choice, individuals will personally balance risks and benefits of personal genomic data sharing (Karczewski et al. 2012). Nevertheless, inherent limitations on controllability of genomic data, once publicly shared, cast doubts on this argument. As it has been noted, “…the notion of consenting to research use of data loses meaning when the use can involve many unknown researchers and uses in perpetuity. Such open-ended use of data renders the well-established right to withdraw consent to collection and use of personal data for research meaningless” (Vayena et al. 2013). Moreover, the evolving nature of genomics research makes it hard to predict the breadth of information that could be extracted from sequenced data in the future and magnitude of risks associated with such disclosures.

Some have stressed on the necessity of communication of methodological limitations of research carried out by some personal data sharing initiatives to their participants and pointed to the current shortcomings in accomplishing this task (Janssens and Kraft 2012). This imperative should be extended to the provision of an accurate account of limitations associated with governance of personal genomic data sharing projects and safeguards to protect rights of participants. To this end, it is suggested to employ adequate communication tools in order to inform potential participants regarding such limitations. Thereby, participants would receive a fair opportunity to consider existing limitations and risks in the face of the highlighted benefits of data sharing for research. This approach will ultimately lead to a higher level of transparency and trust among participants. In addition, the evolving potentials of genomics and bioinformatics render the risks of re-identification and privacy breaches as moving targets, requiring an ongoing investigation of the sufficiency of the pertinent legal and ethical safeguards in place.

Furthermore, given the hereditary nature of genomic data, personal genomic data sharing should not be portrayed as merely a personal decision regardless of the potential ramifications for family members. As the Presidential Commission noted within the recent report, “Respect for persons supports giving persons the opportunity to share their whole genome sequence information for scientific advancement, subject to strong baseline privacy protections. At the same time, individuals have a responsibility to safeguard their privacy as well as that of others, by giving thoughtful consideration to how sharing their whole genome sequencing data in a public forum might expose them to unwanted incursions upon their privacy and that of their immediate relatives. To be indifferent to the implications of disclosure of sensitive data and information about one’s self is to act irresponsibly” (Presidential Commission for the study of bioethical issues 2012).

### Endnotes

^a^dbGaP. http://www.ncbi.nlm.nih.gov/gap. Accessed 21 March 2015

^b^European Genome-phenome Archive. https://www.ebi.ac.uk/ega/. Accessed 21 March 2015

^c^Free the Data. http://www.free-the-data.org/. Accessed 21 March 2015

^d^Genomera. http://genomera.com/. Accessed 21 March 2015

^e^Patients like me. http://www.patientslikeme.com/. Accessed 21 March 2015

^f^Consent to Research. http://weconsent.us/informed-consent/. Accessed 21 March 2015

^g^Genomes Unzipped. project. http://genomesunzipped.org/project. Accessed 21 March 2015

^h^Sage Bionetworks. http://sagebase.org/. Accessed 21 March 2015

^i^Portable Legal Consent. http://sagebase.org/category/portable-consent/. Accessed 21 March 2015

^j^PLC-CGR documents. https://github.com/plc-cgr/plc. Accessed 21 March 2015

^k^Free the Data. Learn More. http://www.free-the-data.org/learn. Accessed 21 March 2015

^l^Free the Data. Free my Data. http://www.free-the-data.org/freemydata. Accessed 21 March 2015

^m^Free the Data. For Clinicians. http://www.free-the-data.org/clinicians. Accessed 21 March 2015

^n^Free the Data. About Us. http://www.free-the-data.org/about. Accessed 21 March 2015

^o^Personal Genome Project. http://www.personalgenomes.org/. Accessed 21 March 2015

^p^1000 Genomes. http://www.1000genomes.org/. Accessed 21 March 2015

^q^Participant Information Form GNZ. http://genomesunzipped.org/wp-content/uploads/2010/10/gnz_consent_phase2launch_101013.pdf. Accessed 21 March 2015
